# Compliance of Type 2 Diabetes Applications to International Guidelines: Protocol for a Quantitative App Assessment

**DOI:** 10.2196/48781

**Published:** 2024-01-31

**Authors:** David Rebus, Andrew Iskander, Felicia Deonarine, Asad Almas, Darren Rattigan, Patrick Henn, Kayode Philip Fadahunsi, John O'Donoghue

**Affiliations:** 1 Faculty of Medicine University College Cork Cork Ireland; 2 Department of Medicine Royal College of Surgeons Ireland Dublin Ireland; 3 ASSERT Centre University College Cork Cork Ireland; 4 Department of Primary Care and Public Health Imperial College London London United Kingdom; 5 Malawi eHealth Research Centre University College Cork Cork Ireland

**Keywords:** diabetes, mobile apps, Mobile Apps Rating Scale, mHealth, mobile health, diabetes application, application, chronic condition, monitoring, accuracy, safety, tool, assistance, treatment, management, type 2 diabetes

## Abstract

**Background:**

Diabetes is among the most common chronic conditions people live with across the world. While it can be managed to a substantial degree, it can result in significant complications. As such, easy access to accurate tools to aid diabetes management is useful in minimizing these complications. Mobile apps are highly accessible and widely used, but there is a gap in the literature examining their compliance with medical guidelines.

**Objective:**

The aims of this study are to develop the Analysis of Diabetes Apps (ADA) checklist to evaluate apps’ compliance to guidelines set by the International Diabetes Federation (IDF) on the treatment and management of type 2 diabetes; to assess type 2 diabetes apps in the Apple App Store and the Android Google Play Store, and their compliance with international guidelines using the ADA framework; and to compare the novel ADA checklist against both the Mobile App Rating Scale (MARS) tool kit and app ratings for each store.

**Methods:**

We will develop a checklist based on the “IDF Clinical Practice Recommendations for Managing Type 2 Diabetes in Primary Care.” Type 2 diabetes apps will be scraped from 6 countries’ app stores using web scraping tools. These countries include Australia, Brazil, India, Nigeria, the United States, and the United Kingdom, which were selected based on the largest population of English-speaking people in each continent. The apps will be searched on the web-based scraper using the search terms “blood sugar,” “diabetes,” “glucose level,” “insulin,” “sugar level,” and “type 2 diabetes.” Apps will be excluded if they are paid or are not in English. The apps will be assessed using the ADA checklist to evaluate their compliance to the international diabetes guidelines. Once scored, the results will be analyzed with descriptive statistics. The most popular apps will be further analyzed using the MARS tool kit. The ADA checklist scores will then be compared to both the MARS tool kit score and app ratings for each store.

**Results:**

The ADA checklist developed based on the IDF guidelines focuses on general information, risk factors, diagnosis, pharmacology, lifestyle modification, glycemic recommendations, and medications. The initial stress testing of the protocol resulted in 173 included apps. This will vary in the final search as the app stores are constantly changing.

**Conclusions:**

The protocol presents the development of a checklist to investigate the compliance of type 2 diabetes apps with international guidelines. The checklist will hopefully form the basis of a scoring system for future research on compliance of mobile apps with international guidelines. High standardization of the ADA checklist will make it a robust tool for people with diabetes and their health care providers alike in assessing type 2 diabetes apps in the future.

**International Registered Report Identifier (IRRID):**

PRR1-10.2196/48781

## Introduction

Type 2 diabetes mellitus (T2DM) is a chronic condition that is prevalent worldwide with its prevalence rising in low-, middle-, and high-income countries [1-5]. In the United Kingdom alone, around 4.7 million people were diagnosed with T2DM in 2019 representing about 7% of the population [6,7]. The Centers for Disease Control and Prevention (CDC) reported that 34.2 million people had T2DM in the United States in 2020 [2].

For individuals who are living with T2DM, there are significant risks of complications including cardiovascular and peripheral vascular damage. In the United Kingdom, T2DM causes 20% of strokes [8]. Individuals living with T2DM are 2.5 times more likely to have a myocardial infarction with 25% of patients arriving in a hospital for a stroke, myocardial infarction, or heart failure also having preexisting diabetes [6]. However, the complications also go beyond circulation, and there can be damage to the peripheral nerves as well. Foot ulcers are a common complication of T2DM. These can lead to foot amputations at much higher rates than those without T2DM. This significant change to a person’s life can also increase their risk of death, with 40% of people who have had a major amputation dying within 5 years post surgery [6]. Other damages can be caused by the production of advanced glycosylated end products. Retinopathy is a common complication causing blindness. Nephropathy is also a common complication that can lead to kidney failure and death [6].

Diabetes treatment is multifaceted. There are multiple drug therapies as well as tertiary preventive measures that are required to minimize the complications of the disease [9]. Some of these measures including blood sugar monitoring and healthy nutrition are supported through mobile health (mHealth) apps [10]. The use of mHealth apps has shown benefits in the past for T2DM. In 1 paper, there was better control of glycemic indicators [11]. One study showed a reduction in hemoglobin A_1c_ in those who used diabetes apps regularly compared to a control population [12]. Previous research has shown better control of glycemic indicators for individuals who use mHealth applications for the management of their disease compared to those who use other methods [8,10,12-14]. However, more research is required to determine the quality of available apps including the accuracy of the information content.

In the past, mHealth apps for T2DM have been evaluated for their functionality, but not for the quality of the information provided in the apps. mHealth apps are often analyzed through the prism of app review criteria such as the Mobile App Rating Scale (MARS) [15]. In the MARS tool kit, apps are rated based on engagement, functionality, aesthetic information, and subjective opinion. Each of these categories is broken down further into questions whose answers range from inadequate to excellent. While this is good for the analysis of apps across many genres, it does not focus on the quality of specific recommendations on the management of T2DM from the guidelines.

Every country has its own specific guidelines for the management of T2DM. Each varies slightly in recommendations such as the diet preference or the pharmacology. The largest area of variation in guidelines between countries comes in the form of diet and exercise [9]. Each of the countries’ guidelines suggests healthy eating, weight loss, as well as moderate exercise. Some countries go into more detailed suggestions for diets for instance in the United States, it is suggested that a weight loss diet of vegetarianism or low carbs is best, whereas in other countries, a Mediterranean diet is recommended. Recommended diets include DASH, Mediterranean, Nordic, or vegetarianism. The DASH diet is comprised of a meal plan with well-balanced meals with lower levels of fat, sugar and sodium in the diet [16]. Similarly, each country recommends at least 150 minutes of moderate exercise a week with variations in the type of recommended exercise. For instance, yoga is recommended in India’s guidelines [17]. The differences in the national guidelines can lead to confusion for both users as well as app designers when apps are released in multiple countries [9].

In this review, we will analyze T2DM apps in a selection of countries with the highest population of English speakers in each continent, including Australia, Brazil, the United Kingdom, India, Nigeria, and the United States. As a result, this paper will evaluate the selected apps based on the recommendations of the International Diabetes Federation (IDF), which is a combination of hundreds of the world’s national diabetes associations. As a result, the IDF’s guidelines form a clear source of recommendations for the management of patients with T2DM across the world. Therefore, we aim to assess the content of selected apps for T2DM based on the IDF’s diabetes guidelines [9].

In addition, the result of the content assessment will be compared to the users’ rating and the result of another assessment of the same apps using MARS, which is a validated scale. The aim of this comparison is to examine concordance or otherwise between apps’ compliance with guidelines, users’ ratings, and assessment scores based on validated scales.

## Methods

### Development of the Analysis of Diabetes Apps Checklist

The checklist (Multimedia Appendix 1) covers key areas of T2DM care. The checklist was developed based on the sections of the “IDF Clinical Practice Recommendations for managing Type 2 Diabetes in Primary Care” [9]. The Analysis of Diabetes Apps (ADA) checklist focuses on general information, risk factors, diagnosis, pharmacologic treatment, lifestyle modification, glycemic recommendations, and medications. The first section focuses on health education. It asks if the app informs the users of the risk factors as listed in the IDF guidelines. The apps gain points depending on what is mentioned. The checklist is useful in that it provides a qualitative manner of ranking apps based on their information content. Information on diabetes diagnosis will also be assessed based on various diagnostic tests and laboratory values used by different systems. Glycemic targets will also be evaluated for accuracy as well as the different units used. Pharmacologic treatment is key for the management of T2DM and will also be evaluated. The final and largest section is the reduction of risk. This includes diet recommendations, exercise strategies, and other lifestyle modifications.

### Sources and Search Terms

Both the Google Play Store as well as the Apple App Store will be searched, using a web scraping tool. Google Play Store and Apple Apps Store are the largest app stores on the market for the public. Both stores will be searched across 6 countries including Australia, Brazil, the United Kingdom, India, Nigeria, and the United States, which are countries that have the highest population of English speakers in their respective continents [18-22]. Although English is not the official language of Brazil, it has the highest population of English speakers in South America due to its large population. The inclusion of countries from all continents is intended to achieve a broad geographic coverage, which allows for increased generalizability of the study findings. Limiting the search to only 1 country per continent is aimed to reduce duplication as the same or similar apps are likely to be available across different countries, especially in the same continent.

The search terms to be used are blood sugar, diabetes, glucose level, insulin, sugar level, and type 2 diabetes. These terms were selected after a series of preliminary screenings. The search terms that yielded the most relevant apps were chosen. Each search term will be entered into a web scraping tool to collect apps.

### Eligibility Criteria

This review will collect a list of apps that are related to T2DM so that they can be evaluated in terms of their compliance with the recommendations of the IDF. The inclusion criteria for the apps are that (1) the content of the apps is in English; (2) the apps are freely downloadable—that is, no payment is required for downloading or using the apps; (3) apps that focus on the aspects of self-management of T2DM such as diet, physical activity, blood sugar monitoring, and foot care; and (4) apps that are compatible with iOS and Android mobile platforms. Evidence from the literature suggests that T2DM is a condition that is prevalent among people with lower socioeconomic status [23]; hence, fees might limit the use of apps among people with T2DM.

Apps will be excluded based on the following criteria: (1) generic lifestyle apps that are not focused on T2DM; (2) apps requesting fees for download or use; (3) apps in languages other than English; and (4) apps with missing or incorrect information will also be removed as it will be difficult to locate and download them from the app stores. The expected data for each app on the scraping platform include the name of the app, the URL, the title, the star rating score, the last update, the publishing date, the genre, and the number of reviews.

### App Selection

The top 30 apps from each keyword search result will be collated due to the limits of the scraping platform. Using the 6 identified search terms for each of the 6 countries will result in 36 different searches and a maximum of 1080 apps. The eligibility criteria relating to cost, language, and compatibility of the apps will be automatically applied using the filters on the scraping platform.

**Figure 1:**
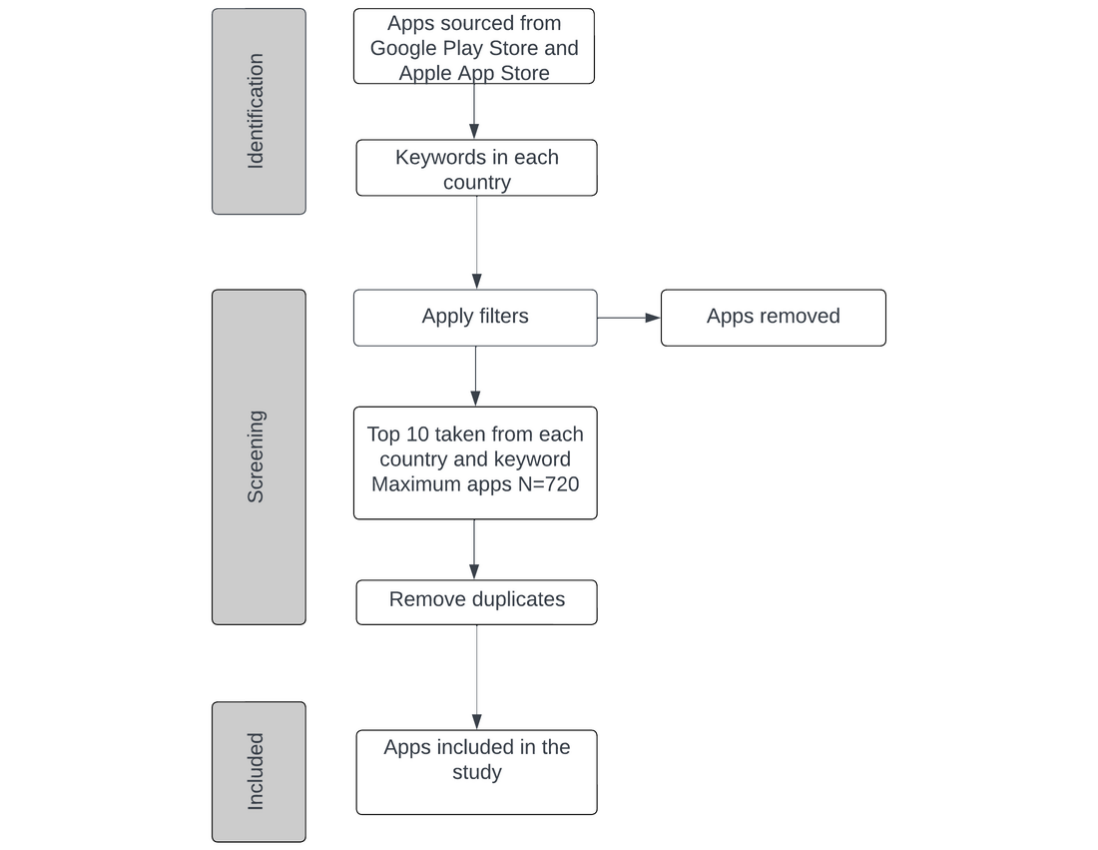
App selection flowchart.

Once the app list is collated, the top 10 apps from each store on each search will be selected with a maximum number of 720 apps. The ranking of apps will be based on the search results with apps higher in the search result being higher on the list. The apps will not be ranked by star rating score or the number of downloads but by whichever method Apple and Google Play sort the apps as this is more representative of what a user would see when searching for a diabetes app [24]. After the compilation of the top apps, duplicates will be removed. A PRISMA (Preferred Reporting Items for Systematic Reviews and Meta-Analyses) diagram showing the proposed scraping method is presented in Figure 1. A similar app scraping methodology has been used in previous assessment studies [25,26].

### Apps Evaluation

The included apps will be evaluated within 6 months of first scraping using the novel ADA checklist (Multimedia Appendix 1) developed by the authors from IDF clinical practice recommendations for managing T2DM in primary care [9]. A total of 2 evaluators will independently assess each app based on the checklist. Disagreement will be resolved by discussion between the 2 evaluators [26].

The assessment data will be analyzed by descriptive statistics. As the data from the scoring system are ordinal, they will be compared via frequency distributions, median, and range. This will be used for each of the checklist items to determine whether there are associations or patterns that are visible.

### Comparison With Other Rating Systems

Additionally, 12 of the apps will be evaluated using the MARS tool kit. The top app for each country under the keyword diabetes will be selected from both the Google Play Store and the Apple App Store. If there is duplication, the next app on the list will be selected. The results of the app assessment based on MARS will be compared to the results of the app assessment based on the ADA checklist to determine whether there are concordance or correlations. Further comparison will be done to ascertain whether there is a correlation with the star rating of the app stores. We would like to assess whether the score from the ADA checklist has a correlation with scores from MARS and the app stores’ star rating. To test for correlation, Spearman rank correlation analysis will be used to compare the ADA checklist’s total score to both the MARS total mean score and the app stores’ star rating for the 12 selected apps.

### Ethical Considerations

No ethics approval is required for this paper as it will not involve animal or human participants and all data to be collected are publicly available.

## Results

The ADA checklist (Multimedia Appendix 1) developed based on the IDF guidelines focuses on general information, risk factors, diagnosis, pharmacologic treatment, lifestyle modification, glycemic recommendations, and medications. The initial stress testing of the protocol resulted in 173 included apps. This will vary in the final search as the app stores are constantly changing.

## Discussion

### Overview

This is the first time, to the best of our knowledge, that an mHealth app assessment checklist is being developed based on medical guidelines and tested on apps in the mobile app stores. The potential uses of this ADA checklist address a number of issues related to the quality of mHealth apps for T2DM. Primarily, it would provide patients with T2DM and their health care providers with a reliable tool by which they can assess whether diabetes apps provide information based on medical guidelines. The lifestyle changes required as part of diabetes care are extensive and the health consequences of failing to make these changes in a timely and appropriate manner can be severe. Due to the lack of filters in mobile app stores related to compliance with guidelines, it is of the utmost importance that if patients are reliant on apps such as those to be evaluated by this protocol, they are able to avoid those that would mislead them. This new checklist may be useful for further assessment of diabetes apps based on their compliance with medical guidelines. The ADA checklist will allow for more trust and transparency regarding diabetes apps that are currently used, as well as future applications.

Furthermore, it would be of interest to assess if users’ star ratings in the app store correlate with app quality based on the novel ADA checklist, as most prospective users might have been relying on user ratings while choosing their apps. Users’ star ratings, as a metric, are open to a number of biases that could potentially reduce its correlation to app quality [27,28]. For example, user reviews may be made on the basis of user interface, aesthetics, or the invasiveness and frequency of any advertisements the app displays [27,28]. Previous studies have compared users’ star ratings with MARS, an analysis of whether app ratings correlate to compliance with IDF guidelines in this study will further assess the use of users’ ratings. On the other hand, comparison with MARS could help to assess whether apps that do well on assessment with a generic mHealth app assessment tool also follow medical guidelines. Irrespective of the results of this comparison between the generic app assessment tool (MARS) and the guidelines-based ADA checklist, both are likely to become complimentary as they focus on different aspects of mHealth apps.

This novel methodology for assessing apps based on medical guidelines can be translated to apps for other conditions such as hypertension, hypercholesterolemia, and atrial fibrillation. While the aspects of the checklist will change between different conditions, the checklist could be used as a baseline framework in future studies, especially with other chronic conditions.

### Limitations

The proposed methodology has a number of limitations. First, the app selection process is dependent on the order in which the apps are listed on the search results on both the Google Play Store and the Apple App Store. These algorithms are subject to change at any time without warning, and these changes would likewise be unbeknownst to users. For the purposes of this protocol, this unknown ordering process will influence which apps will be included or excluded in our evaluation, as only the first 30 apps from each set of search results will be included for evaluation, excluding duplicates. This may affect the reproducibility of the results, given the potential for the order of the app listings to change on search results without notification or warning. However, this approach was deemed to be the most objective and unbiased way to select the apps that would be evaluated as these are the apps that are seen first by the users. While analyzing apps based on the number of downloads may be useful, it skews the results of the search to older apps that may not be up to date on guidelines.

Another limitation of this protocol is that only free apps will be evaluated. This is a reasonable exclusion criterion for the purposes of this evaluation, as it would ultimately provide useful information for patients with diabetes in search of a medically accurate and useful app but who are not willing or able to pay for one. However, this also means that the results of this evaluation will not be generalizable across all diabetes apps; it may well be that many purchasable apps, or components of free apps that require in-app purchases, are better than those that would be included in this evaluation based on the criteria described above.

In addition, comparison with the apps’ ratings may be problematic. The average score on a 5-star rating system may be skewed by the variability in the number of reviews given. Because we intend to evaluate many apps, there is bound to be a wide range in a number of reviews. As such, apps with few reviews are likely to have less reliable average ratings compared to those with high numbers of reviews. Additionally, this would not at all account for app developers who use methods to inflate their respective app store reviews. Controlling the number of reviews may be a helpful step in reducing the bias this could introduce, though no obvious or reliable method to reduce this bias exists.

### Conclusions

There are hundreds of thousands of health-related apps in the app stores. Such apps have been assessed previously from different perspectives, including information quality, reliability, interface, and efficacy. However, there have been no tools to assess apps from a clinical guideline perspective. This methodology developed in this research will demonstrate how to assess the adherence of mHealth apps to clinical guidelines.

## Appendix

Multimedia Appendix 1 ADA checklist to be used in protocol.

## Abbreviations

ADA: Analysis of Diabetes Apps
CDC: Centers for Disease Control and Prevention
IDF: International Diabetes Federation
MARS: Mobile App Rating Scale
mHealth: mobile health
PRISMA: Preferred Reporting Items for Systematic Reviews and Meta-Analyses
T2DM: type 2 diabetes mellitus

## Data Availability

Data sharing is not applicable to this article as no data sets were generated or analyzed during this study.
