# Supplementary material for: Compliance of Type 2 Diabetes Applications to International Guidelines: Protocol for a Quantitative App Assessment
Source: JMIR Res Protoc. 2024 Jan 31;13:e48781. doi: 10.2196/48781 (PMC10867742; doi:10.2196/48781)
Supplement: Multimedia Appendix 1 [file resprot_v13i1e48781_app1.pdf]

Category	Checklist Item	Definition	Criteria	Mars Comparison
Health education	High BMI	Does the app provide education or mention this risk factor related to diabetes type 2?	No = 0<br>Yes = 1	Section D 15 Is app content correct, well written, and relevant to the goal/topic of the app
	Age <45			
	Gender/ sex			
	Family history			
	Hypertension			
	Inactivity			
	Daily consumption of fruit and veg			
Diagnosis	Fasting blood glucose of 7mmol/ 126mg/dl	Does the app include this method of diagnosing type 2 diabetes?	No = 0<br><br>Yes but the measurement or units are incorrect = 1<br><br>Yes but no measurements or units are included = 2<br><br>Yes and including measurements = 3	Section D 15 Is app content correct, well written, and relevant to the goal/topic of the app
	2hr OGTT ≥ 200mg/dl 11.1mmol/L			
	Hba1c ≥ 48mmol.mol 6.5%			
Glycemic targets	HbA1c of <7% (53 mmol/mol)	Does the app include this the HbA1c value and its variants	No = 0<br><br>Yes but the measurement or units are incorrect = 1<br><br>Yes but no measurements or units are included = 2<br><br>Yes and including measurements =	Section D 15 Is app content correct, well written, and relevant to the goal/topic of the app
	Always avoid blood glucose below 3mmol/l (54mg/dl)			
	Special populations may not use the same guidelines			

	There use range of 7.5% to 8% (58-64mmol/mol		3	
Drug Information	First line suggestions	Does the app suggest first line medications that are indicated by the IDF guidelines for treatment?	No = 0<br>Yes = 1	Section D 15 Is app content correct, well written, and relevant to the goal/topic of the app<br>Section D 15 Is app content correct, well written, and relevant to the goal/topic of the app
	Metformin	Does the app provide information about metformin being the first choice in the treatment of type 2 diabetes?		
	Dosage	Does the app suggest contacting the user's physician/ pharmacist or reading the medication packaging for information regarding the dosage, contraindication and side effects?		
	Contraindications			
	Side effects			
	Combination therapy	Does the app educate the user about the possibility of these therapies?		
	Initial insulin therapy			
	Dual therapy			
	Triple therapy			
Reduction of risk factors	Aerobic exercise	Does the app mention moderate aerobic exercise	No = 0<br>Yes but no details = 1<br>Yes + details	Section D 15 Is app content correct, well written, and

		(E.g. walking) for at least 150 minutes per week?	such as timing = 2	relevant to the goal/topic of the app
	Resistance training	Does the app mention intense physical training or resistance training (E.g. yoga and weight lifting)?		
	Weight Loss training	Does the app mention intense physical training for the purposes of weight loss for at least 275 minutes per week?		
	Consult a dietitian	Does the app recommend consulting a dietician?	No = 0<br>Yes = 1	
	Calorie deficit diet	Does the app discuss any of these types of diets?		
	Mediterranean diet			
	Keto diet			
	High fiber, low glycemic index/ 3-5 portions of vegetables and fruit, fish, grains and monosaturated fats			
	Bariatric surgery	Does the app mention the use of bariatric surgery to aid in weight loss for the treatment of		

		type 2 diabetes?		
	Smoking	Does the app recommend cessation of smoking?		
	Alcohol	Does the app mention that those with type 2 diabetes should not exceed 1-2 units of alcohol per day?		
Score				
